# The Lymphatic System in Breast Cancer: Anatomical and Molecular Approaches

**DOI:** 10.3390/medicina57111272

**Published:** 2021-11-19

**Authors:** Gianfranco Natale, Michael E. J. Stouthandel, Tom Van Hoof, Guido Bocci

**Affiliations:** 1 Department of Translational Research and New Technologies in Medicine and Surgery, University of Pisa, 56126 Pisa, Italy; 2 Museum of Human Anatomy “Filippo Civinini”, University of Pisa, 56126 Pisa, Italy; 3 Department of Human Structure and Repair, Ghent University, 9000 Ghent, Belgium; Michael.Stouthandel@UGent.be (M.E.J.S.); Tom.VanHoof@UGent.be (T.V.H.); 4 Department of Clinical and Experimental Medicine, University of Pisa, 56126 Pisa, Italy; guido.bocci@unipi.it

**Keywords:** breast cancer, lymphatic system, lymphatic metastasis, sentinel lymph node, anticancer drugs

## Abstract

Breast cancer is one of the most important causes of premature mortality among women and it is one of the most frequently diagnosed tumours worldwide. For this reason, routine screening for prevention and early diagnosis is important for the quality of life of patients. Breast cancer cells can enter blood and lymphatic capillaries, then metastasizing to the regional lymph nodes in the axilla and to both visceral and non-visceral sites. Rather than at the primary site, they seem to enter the systemic circulation mainly through the sentinel lymph node and the biopsy of this indicator can influence the axillary dissection during the surgical approach to the pathology. Furthermore, secondary lymphoedema is another important issue for women following breast cancer surgical treatment or radiotherapy. Considering these fundamental aspects, the present article aims to describe new methodological approaches to assess the anatomy of the lymphatic network in the axillary region, as well as the molecular and physiological control of lymphatic vessel function, in order to understand how the lymphatic system contributes to breast cancer disease. Due to their clinical implications, the understanding of the molecular mechanisms governing lymph node metastasis in breast cancer are also examined. Beyond the investigation of breast lymphatic networks and lymphatic molecular mechanisms, the discovery of new effective anti-lymphangiogenic drugs for future clinical settings appears essential to support any future development in the treatment of breast cancer.

## 1. Introduction

Breast cancer represents one of the most important causes of premature mortality among women and it is one of the most frequently diagnosed neoplastic diseases worldwide [1]. A large variety of risk factors for this severe pathology has been recognised by epidemiologic studies: race, ethnicity, family history of cancer, and genetic traits, as well as increased alcohol consumption, physical inactivity, exogenous hormones, and female reproductive factors [2,3]. Unlike tumours of internal organs, breast cancer is one of the types of cancer most often described since ancient times because of its appreciation through palpation and visibility through the skin, with ulcerative lesions and bleeding [4]. Together with imaging techniques, self-examination still represents an important surveillance step in the routine screening for the early diagnosis of breast cancer and prevention of tumour growth and metastasis [5].

Unfortunately, breast cancer cells can enter blood and lymphatic capillaries. Thus, molecular mechanisms allow this tumour to metastasise to the regional lymph nodes in the axilla and to both visceral and non-visceral sites. In addition to this, secondary lymphoedema is another important issue for women following breast cancer surgical treatment or radiotherapy. The lymphatic system, including lymphatic vessels and lymph nodes, plays a pivotal role in both tissue–fluid balance and immune cell trafficking and response. In spite of its importance, the gross anatomy of this system has been neglected for a very long time. Indeed, unlike the cardiovascular system, the lymphatic network is characterised by almost invisible and fragile vessels which transport lymph, a clear and colourless fluid [6].

**Figure 1:**
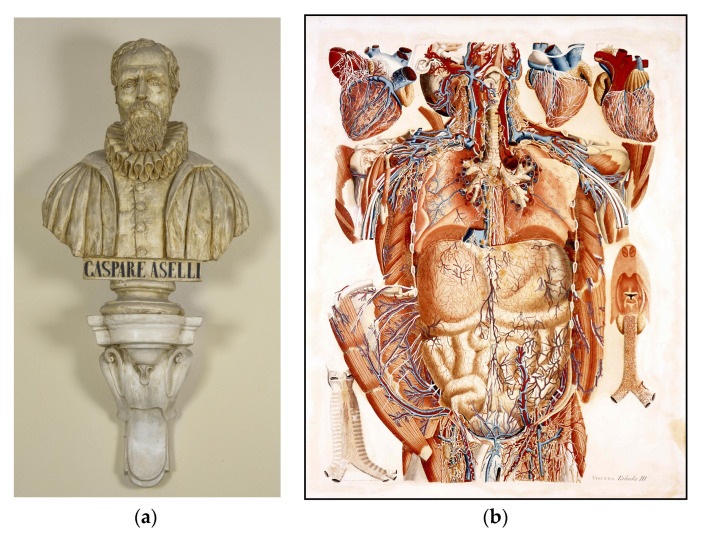
(**A**) Plaster bust of Gaspare Aselli. Museum of Human Anatomy “Filippo Civinini”. University of Pisa. (**B**) *Tabula III-Viscera* (Paolo Mascagni. *Anatomiae universae icones*) [14]. This plate illustrates the lymphatic network in thoracic, axillary and inguinal regions. Mascagni’s Gallery of the Museum of Human Anatomy “Filippo Civinini”. University of Pisa.

When reviewing the historical identification of the lymphatic system, it is not surprising to find that the lymphatic system was initially discovered by chance, since anatomists encountered technical difficulties in discovering and demonstrating this system. In 1622, the Italian anatomist Aselli (1581–1625) (Figure 1A) casually showed gut lymphatics in well-fed dogs, because of the milky appearance of the lymph due to its lipid content. For this reason, they are still named lacteals [7]. On the contrary, peripheral parts of the lymphatic system are translucent and contain a colourless liquid (lymph) [6,8]. They therefore need to be contrasted in order to distinguish these tiny vessels with the naked eye, making contrast injections a key part of lymphatic research. Thus, in the eighteenth century, injection techniques were developed to identify the lymphatic network and several scientists were able to describe its gross anatomy [9,10]. It was only in the 1690s when Nuck discovered that mercury could be used as a contrast agent for the lymphatic system and anatomists were able to visualise the lymphatics that were not filled with chyle [11]. With the aid of this mercury injection technique, the lymphatics of the breast were first described by Cruickshank in 1786 but he did not present any figures to show them [12]. In 1787, the Italian anatomist Mascagni published a very detailed work on the lymphatic system, *Vasorum lymphaticorum corporis humani historia et ichnographia* [13]. This system appeared also in his posthumous work *Anatomiae universae icones*, including the axillary region [14] (Figure 1B), but the lymphatics of the breast were not presented yet.

The first depiction of the lymphatics of the breast was provided by Sappey in 1874, again by using a mercury injection approach [15]. This landmark work of Sappey contained such detail that it has long since been regarded as flawless and it is currently still a primary source for information on the lymphatic system for both anatomists and clinicians [16]. After Sappey, Romanian anatomist Gerota developed a contrast agent based on blue oil paint to replace the toxic mercury injections [17], while Poirier and Cuneo provided an overview of the lymphatic drainage of the breast in 1902 [18]. This new contrast agent only advanced for a short distance inside the lymphatics however and it was therefore necessary to apply this technique to child or foetal cadavers [17,19]. Seeing as the breasts are not yet developed in children, additional information about the lymphatic drainage of the breast was not available until Suami and his colleagues developed a new injection technique that could be applied in adult cadavers and that also allowed radiographic imaging in 2005 [20,21]. The latest anatomical depictions of the lymphatic system of the breast are derived from Suami’s methodology and they have made a meticulous effort to map the superficial lymphatics of the arm and breast in recent years, either by using lead oxide or indocyanine-green-based injection solutions [22,23,24,25].

More in depth, Suami et al. [16] and Wai [26] published interesting historical articles dedicated to the description of breast lymphatic vessels, from past anatomical studies to modern techniques. In spite of these studies, the anatomy and physiology of breast lymphatics need to be re-examined and better elucidated to provide further knowledge. This body of information is important among pathologists, surgeons, oncologists, pharmacologists, radiologists, and radiotherapists who deal with breast cancer prevention and therapy [27]. Not surprisingly, the anatomy of the lymphatic system appears different and changes its organization according to the organ where this network is developed. Thus, apart from meningeal lymphatics [28], in the central nervous system, where a classical lymphatic network is not recognised, the intriguing glymphatic pathway has been described as an alternative system for waste clearance [28,29].

In recent times, new anatomical and physiological concepts have been developed to better understand the lymphatic system. First of all, the novel anatomical concept of “lymphosome”, that is skin superficial lymphatic territory, was introduced to describe how the lymphatic vessels in a particular region connect to the same subgroup of regional lymph nodes, as well as the anatomical relationship between the perforating lymphatic vessels and arteries. Since the normal anatomy of the lymphatic system is important for predicting which lymph nodes may be affected by metastases after a primary tumour, topographic knowledge can help this approach. This concept is also relevant to understand the secondary lymphoedema occurring after lymph node removal in cancer patients [30].

Collecting lymphatic vessels are provided with classical valves and recently they were also found to be surrounded by specialised lymphatic muscle cells. The muscular segment between two valves causes contraction and behaves like an autonomous pump that drives the unidirectional transport of the lymph to lymph nodes. This specialised segment was named “lymphangion”. The mechanisms of this lymphatic contractility seem to depend on different factors, including nitric oxide and the dynamics of intracellular calcium. After a contraction, nitric oxide induced by lymph flow over lymphatic endothelial cells triggers muscular relaxation and vessel dilation, which allows for lymphangion filling [31,32,33,34,35].

How cancer cells metastasise is still a matter of debate. Different patterns of spread from the primary tumour to the systemic circulation have been suggested. Some malignant cells seem to gain access directly at the site of the primary tumour. Since tumours have elevated interstitial fluid pressure, this can facilitate the entry of cancer cells through endothelial cells of lymphatic capillaries. Furthermore, the discovery that tumours can induce angiogenesis, that is the formation of new blood or lymphatic vessels, confirmed this hypothesis and the term “lymphovascular invasion” was coined for this concept [36]. In the case of breast cancer, the majority of vessels involved in this invasion were shown to be lymphatic. In line with this, radical mastectomy for the surgical treatment of breast cancer represented the best approach. It was also shown that cancer cells can enter the systemic circulation through the sentinel lymph node, where a premetastatic niche was described. In this case, cancer cells proliferate in this lymph node, where they encounter different types of vessels, including dilated high endothelial venules, and newly formed blood and lymphatic capillaries induced by vascular endothelial growth factors. Thus, cancer cells enter the blood circulation to spread. Rather than at the primary site, breast cancer cells seem to gain entrance to the systemic circulation mainly through the sentinel lymph node and the biopsy of this fundamental indicator can influence the axillary dissection during the surgical approach to the pathology. Nevertheless, the role of axillary lymph nodes in seeding distant organ metastases remains to be elucidated [6,35,36,37,38,39,40,41].

However, cancer cells can also disseminate from primary breast cancer via blood vessels after intensive neo-angiogenesis (stimulated by pro-angiogenic growth factors such as VEGF-A) which is thus called haematogenous spread. New angiogenic blood vessels are usually abnormal, without pericytes and with numerous fenestrations. These characteristics permit intravasation and the spread of circulating cancer cells (CTC) [42]. As an example, the pulmonary metastases of breast cancer find in the hematogenous mechanism their most common route of spreading [43]. Moreover, in breast cancer, CTC quantification has been used as a prognostic and predictive biomarker to guide treatment in the metastatic disease [44]. The hematogenous spread of cancer cells does not depend only on tumour microenvironment and angiogenesis, but also on the characteristics of cancer cells. Recently, Kalinkova and colleagues demonstrated that a decreased methylation in the SNAI2 and ADAM23 genes was associated with the de-differentiation of breast cancer cells and their haematogenous dissemination [45]. Finally, recent data suggested that clusters of circulating tumour cells enriched with stromal cancer-associated fibroblasts in breast cancer patients augmented their hematogenous metastatic potential [46].

Anyhow, the anatomy and biopsy of the sentinel lymph node in breast cancer represent an important preoperative item to be considered. In a retrospectively reviewed study the number of lymphatic pathways and their branching patterns, as well as the number, location, and direction of flow of breast sentinel lymph nodes have been found significantly different when examined under computed tomography–lymphography [6,47].

The presence of lymph node metastasis is one of the most important prognostic factors in breast cancer patients. Due to their clinical implications, the understanding of the molecular mechanisms governing lymph node metastasis in breast cancer is a key aspect for any future research [48]. As an example, two chemokine receptors, CXCR4 and CCR7, have been implicated in regulating the metastatic process. Moreover, the lymphangiogenesis is largely driven by the VEGFR-3 pathway, a tyrosine kinase receptor expressed primarily on the lymphatic endothelium, with VEGF-C and VEGF-D being the main regulators of this pathway. VEGF-C and VEGF-D can promote the formation of intratumoural lymphatic vessels by binding to VEGFR-3, and they can also induce morphological changes in peritumoural lymphatics that support the entry of tumour cells into lymphatic vessels. In transplantation mouse models, VEGF-C increases intratumoural lymphangiogenesis and the incidence of lymph node metastasis. Furthermore, numerous studies show that elevated VEGF-C is reported in 30–40% of breast cancers and it is correlated with a high incidence of lymphovascular invasion, lymph node metastasis and lower disease-free survival. Finally, the identification of lymphatic-specific biomarkers, including podoplanin and LYVE-1, has greatly moved the field of lymphatic metastasis forward [49,50,51]. Thus, the development of animal models recreating the process of lymphatic metastasis is essential, and the incorporation of proteomic and genomic platforms in clinical studies is fundamental to discover potential new lymphatic biomarkers and possible novel therapeutic targets [52].

The present article aims to review new methodological investigations to assess the anatomy of the lymphatic network, as well as the molecular and physiological control of lymphatic vessel function in order to argue how the lymphatic system contributes to disease processes, with particular attention to breast cancer and secondary lymphoedema. New lymphatic imaging and intervention techniques, including the intranodal lymphangiogram, dynamic contrast enhanced magnetic resonance lymphangiography, and lymphatic embolization, have improved the knowledge of lymphatic anatomy. In addition to these novel methods, classical injection techniques can still help anatomists and oncologists in understanding specific clinical conditions. It is critical to identify appropriate biomarkers that can distinguish lymph nodes which are prone to metastasis. In fact, the surgical removal of lymph node metastasis can reduce cancer spreading, but at the same time it can impair systemic anti-tumour immune responses and aggravate lymphoedema. Moreover, the ability of imaging breast lymphatics or identifying lymphatic morphological changes indicative of breast lymphoedema are essential for the quality of life of cancer patients. Again, anatomical knowledge is important to establish surgical lymphatico-venular anastomosis to improve lymphatic drainage.

## 2. Techniques to Characterise Breast Lymphatic Anatomy

After developing their lymphatic injection technique, Suami’s group mainly focused on lymphoedema for their lymphatic injections and as such they were most interested in the superficial lymphatics; meanwhile, a complete overview of superficial and deep lymphatics and their connections could provide even better insights into the lymphatic anatomy. By only presenting their findings on 2D radiographs that do not depict the spatial relations between the lymphatics and the surrounding anatomical structures, it would appear that Suami’s findings can still be improved upon. Suami’s findings conflicted with Sappey’s depictions of the lymphatics of the breast. Sappey showed the subareolar plexus receiving all lymphatics that drain the breast, but Suami and colleagues showed alternative drainage patterns [22]. Recent lymphoscintigraphy studies also suggested that Sappey did not succeed in capturing the full scope of the lymphatics of the breast, since alternative lymphatic drainage pathways were sometimes recorded [53]. It should be noted here that lymphoscintigraphy is only performed in a clinical setting, to map tumour spreading to the sentinel lymph node, thereby only focusing on a small area in the vicinity of the tumour. By means of injecting dye (a radioactive dye in case of lymphoscintigraphy and non-radioactive dyes for lymphangiography) in the vicinity of the tumour, the drainage pattern of the tumour site can be visualised and the sentinel lymph node can be identified for biopsy, because it will be highlighted by the dye [54,55]. Reverse axillary mapping is a special type of lymphatic mapping that can be performed during a surgical intervention. In reverse axillary mapping, dye is injected in the arm, instead of in the breast tissue surrounding the tumour. The goal of this procedure is to trace the dye back to the lymph node(s) that drain the upper arm in order to identify the drainage pathways of the arm. This procedure gives important information for the prevention of lymphoedema of the arm, which can occur as a side effect of surgical intervention, in case no viable drainage pathway for lymph from the arm remains after a surgical intervention. [56] Since both techniques are aimed at patient care, they are concluded as soon as possible to avoid unnecessary exposure to radioactive tracer or a prolonged surgery and they are therefore not suitable for mapping the entire lymphatic network in an area, such as in anatomical studies. Finally, Turner-Warwick even suggested that Sappey had confused the lactiferous duct for lymphatics, based on radioactive colloidal gold injections in patients [57] indicating that these historical atlases, even though immensely valuable as a source for lymphatic knowledge, should not be regarded as flawless.

Considering the fact that the lymphatics are notoriously difficult to visualise, it is no wonder to see that figures of the lymphatics were historically being copied from earlier atlases. For example, a plate of the subclavian lymphatic trunk appearing in Cloquet’s atlas of anatomy (1825) [58] strongly resembles a plate presented earlier in Mascagni’s atlas (1787) [13] and even in 1909, Bartels still used this same plate from Mascagni in his work on the lymphatics [19]. The lymphatics of the breast showed a similar practice of copying previous atlas plates. For example, the plate of Poirier and Cuneo that summarises the lymphatics of the breast and on which injection sites for sentinel lymph node procedures are based was suspected to be composed based on Sappey’s work, supplemented with the authors’ own findings [16], but their findings were based on foetal or child specimens, because they used Gerota’s injection solution. The fact that mercury injections can no longer be performed, thus not allowing the replication of these historical works and the fact that historical anatomists did not have a lot of faith in microscopes yet in the 1800s, as evidenced by entries such as “his observation having been made with very high magnifying powers, are not exempt from a portion of doubt which seems almost inseparable from results so obtained” [59], would suggest that it is high time that the lymphatic system anatomy is re-evaluated. This time, the historical anatomical drawings can even be objectified by using modern medical imaging techniques that will be able to capture the contrast-filled lymphatics in relation to the regional anatomy [60].

Depicting the lymphatic system in relation to bones and soft tissues in the area is crucial for the field of radiotherapy, since treatment planning is performed on patient CT scans that do not show the location of the lymphatics [61,62]. For this purpose, delineation guidelines are available for the lymphatic targets, but they are based on the vasculature (that was shown to be in close proximity to the lymphatics [63]), rather than the actual location of the lymphatic system [63,64]. As such, when spatially accurate representations of the lymphatic system can be provided along with the anatomical structures that can be identified on patient CT scans, the delineation guidelines could be refined and the radiation therapy could become more accurate, meaning that the radiation dose to the target tissues (lymphatics) can be maximised, while the radiation dose to the healthy tissues (heart and lungs) can be minimised, providing better tumour control with fewer side effects [65,66]. It is important to realise that the superficial lymphatics are not the only target of radiotherapy. When metastasis is found in the axillary lymph nodes, the subclavian lymphatic trunk becomes the main target for lymph node treatment, to prevent cancer cells from reaching the bloodstream at the confluence of the thoracic duct with the venous angle [67]. Whereas Sappey’s atlas shows the superficial lymphatics of the breast, the subclavian lymphatic trunk is not included [15]. One of the few original depictions of this subclavian lymphatic trunk can be found in Mascagni’s atlas [13]. Considering that this information is often used in clinical practice, even though it has not been updated in centuries, it seems warranted to revisit/verify this depiction. On the one hand, a more accurate radiation treatment could increase treatment efficacy and prevent long term side effects such as heart disease or secondary lung cancer [68,69]. On the other hand, if lymphatic pathways from the breast and the arm can be separated and the lymphatics that are not associated with the breast can be spared, a more accurate radiation treatment might also have an impact on the incidence of lymphoedema formation in the arm.

In order to obtain the location of the lymphatic system by means of medical imaging, contrast agent will first need to be injected into the lymphatic system. When reviewing the literature on lymphatic injections, there is a clear divide in the injection approach. On the one hand there is the anterograde injection approach, which starts at the smallest lymphatic branches and follows the direction of the unidirectional valves that are present inside the collecting lymphatic vessels. This technique was advocated in the literature and it was also used by Suami et al. [20,21,25]. Even though the technique has shown impressive results on 2D radiographs, the specimens did not prove suitable for medical imaging that allows 3D reconstruction, such as CT or MRI. This delicate technique requires the individual cannulation of translucent, superficial lymphatics in the microscopic range and it takes a great deal of time to complete a specimen, even up to 6 weeks, with 2 people working on it [23]. During this time, putrefaction of the fresh specimens becomes a real issue [70]. The putrefaction process will affect the spatial relations and it prevents taking CT or MRI images after the injections have been concluded. Even with ample opportunities to cool the specimens nowadays, putrefaction of fresh specimens cannot be halted. In the early days of lymphatic research, the hydrogen peroxide injection technique of Suami and colleagues that fills the lymphatics with air bubbles to make them easier to cannulate [20] was not yet available. Instead specimens were placed in water baths containing drained blood to promote putrefaction and obtain air bubbles to fill the lymphatic system so it could be cannulated [71]. If even Suami and colleagues had issues with putrefaction of the specimens, while they started their procedure with fresh specimens that were in good condition, the early anatomists must have had to deal with advanced stages of putrefaction, given the fact that they already started with putrefied specimens and there were no fridges in the 1700s and 1800s to preserve the specimens. As such, it is likely that the injected lymphatic specimens were not in the best condition when the injections were finished and atlas plates were drawn. Finally, considering the putrefaction of fresh specimens, it is also highly unlikely that historical anatomical atlases contain drawings based on a single specimen. Instead, atlas plates are likely made up of several different specimens, maybe even combining data from adult and child/foetal specimens in the same plates. As it is not possible to confirm how many specimens were used for the construction of a certain atlas plate, plates could be based on only one or two specimens and anatomical variability might be severely underrepresented, making a re-examination of these historical works all the more relevant today, now that we have the technological means to objectively capture the results [60].

Based on the flaws of anterograde injection approaches, the other possibility for lymphatic injection, namely retrograde injection, becomes more appealing. Instead of finding all terminal branches of the lymphatics, one could instead cannulate the thoracic duct and inject against the flow of lymph to fill the lymphatic vessels from big to small diameters, rather than the other way around. This could theoretically be performed with only a single cannulation, thereby significantly shortening the injection procedure. The retrograde injection methodology is discouraged in the literature, however, stating that the lymphatic valves will prevent successful retrograde filling. Even though retrograde injections are discouraged, none of the citing authors mention up to which degree retrograde injection was explored before making this statement [20,57,71]. Not only would a retrograde injection approach lower the experimental time significantly, it also allows for a localised dissection to cannulate the thoracic duct at a single point, thus keeping the spatial relations between the subclavian lymphatic trunk (the lymphatic treatment target for radiotherapy) and the surrounding anatomy intact. If successful, the retrograde injection will not only fill the subclavian lymphatic trunk but it will also show how this structure connects to the superficial lymphatics, providing additional valuable information on the lymphatic anatomy. As such, researchers considered exploring a retrograde injection methodology to verify if this was a possibility. To prevent putrefaction and extend the experimental window, embalmed specimens were chosen for this purpose.

Since embalmed specimens had never been used for lymphatic experiments, it was first verified if retrograde injections would be possible in a controlled setting. For this purpose, the thoracic duct was exposed inside the thoracic cavity by removing the ribs, lungs and heart. This approach reveals a long stretch of thoracic duct (20–30 cm) that is easily accessible for cannulation, and lymphatic filling can visually be inspected during the injection procedure. Manual injection with a 50/50 (*v*/*v*) solution of barium sulphate in pre-vulcanised liquid latex confirmed the possibility of retrograde lymphatic injection and it showed that this contrast agent was ideally suited for digital reconstruction of CT images. Lymphatic branches up to 0.23 mm could be visualised on CT and automatic segmentation of the contrast-filled lymphatics (20 min) provided a three-dimensional image that perfectly matched the lymphatics in the dissected specimen. Lymphatics could afterwards be dissected very easily, due to the hardening of the latex that gave the otherwise frail lymphatics a firm composition. This study also provided evidence that embalmed specimens can be used for lymphatic injections. The 0.23 mm diameter of the lymphatics that could be visualised after retrograde injection is in the size range of the subclavian lymphatic trunk, thus indirectly proving that it should be a possibility to retrogradely inject the subclavian lymphatic trunk as well [72]. A final finding of this study was that the manual injection approach was too crude in relation to the delicate lymphatic walls, so a more sophisticated injection method seemed warranted. Instead of manual pressure, gravity-controlled pressure was chosen and a dedicated experimental set up was developed for this purpose.

**Figure 2:**
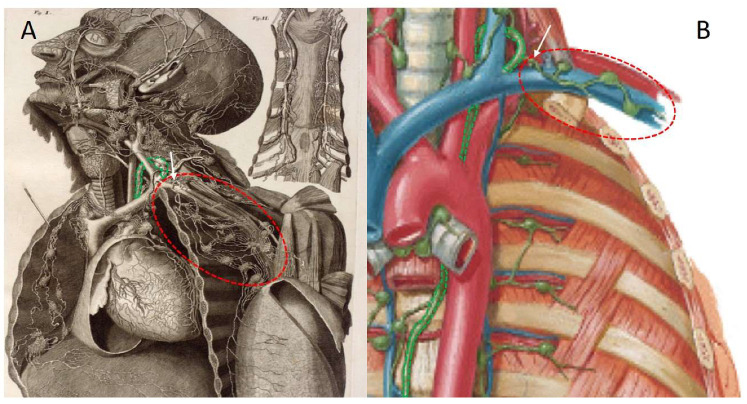
Lymphatic anatomy of the upper quadrant, showing the thoracic duct and the subclavian lymphatic trunk connected to its network of lymph nodes. The thoracic duct is highlighted in green. The subclavian lymphatic trunk (with the main vessel indicated by a white arrow) and the connecting lymph nodes are circled in red. (**A**) This historical plate provides an unobstructed overview of the lymphatics in the area of interest. Note that the subclavian lymphatic trunk does not first connect to the thoracic duct before joining the venous angle. Adapted from the original atlas plate of Mascagni (1787) [13] (Library of Medicine and Pharmacy, University of Pisa). (**B**) In this modern plate, the subclavian lymphatic trunk is much more stylised and it connects directly to the thoracic duct. Adapted from Netter’s atlas of human anatomy [74].

For this experimental set up, the thoracic duct was identified at the confluence with the venous angle. Preparatory dissections in an attempt to retrogradely inject the terminal portion of the thoracic duct revealed an anatomical variant of the subclavian lymphatic trunk that was not depicted in modern day atlases [73,74,75,76]. The subclavian lymphatic trunk did not appear to always join the thoracic duct, before ending in the venous angle. Instead, in several specimens, the subclavian lymphatic trunk directly entered the venous angle, without first joining the thoracic duct. Figure 2 depicts the subclavian lymphatic trunk in both a historical and a modern day anatomical representation to give an overview of the region and to illustrate the difference in detail.

**Figure 3:**
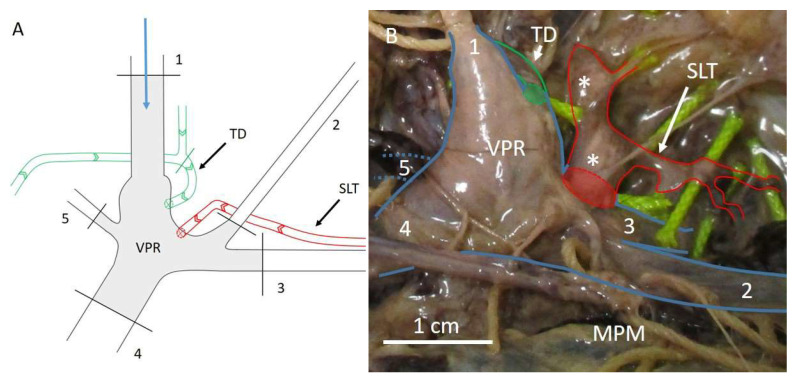
Schematic representation of the venous reservoir (**A**) and the venous reservoir in a cadaveric set up (**B**). The blue arrow in panel (**A**) represents the cannulation site and the flow direction of the contrast agent upon injection. The numbers represent the venous structures that need to be ligated in order to obtain the venous pressure reservoir. Arrows inside the lymphatic vessels in panel (**A**) depict the natural direction of lymph flow. Black lines represent ligation sites. The green line shows the ligation site at the thoracic duct, to prevent contrast agent from advancing into the thorax, instead focusing the built up pressure towards the SLT. Note that the external jugular vein (2) is retracted caudally in panel (**B**), in order to show the SLT that was partially filled with contrast agent as a result of the retrograde injection approach using the venous reservoir. The vertebral vein (5) in panel (**B**) is depicted as interrupted blue lines, because this structure runs posteriorly from the venous pressure reservoir, obscuring it from view. 1 = internal jugular vein, 2 = external jugular vein, 3 = subclavian vein, 4 = brachiocephalic vein, 5 = vertebral vein, MPM = major pectoral muscle, SLT = subclavian lymphatic trunk, TD = thoracic duct, VPR = venous pressure reservoir, * = lymph node. Green lines = thoracic duct, red lines = subclavian lymphatic trunk, blue lines = venous structures in panel (**B**). Adapted from Stouthandel et al. [77].

This finding complicated research, because direct cannulation of the thoracic duct did not automatically guarantee filling of the subclavian lymphatic trunk, since the connection was not always present and there was no clear indication for when this variation would be present. Since the subclavian lymphatic trunk consistently ended in the venous angle, either directly or indirectly after first joining the thoracic duct, it was decided to use the venous angle as a pressure reservoir, rather than the thoracic duct. A dedicated dissection approach was developed that allowed the construction of an intact venous pressure reservoir inside the venous angle. For this pressure reservoir to be functional, all the venous tributaries (internal jugular vein, external jugular vein, subclavian vein, brachiocephalic vein and vertebral vein) had to be ligated. The internal jugular vein was used for cannulation because it provides easy access to the venous angle. Figure 3 shows the venous reservoir schematically and in a cadaveric specimen. Patency of the venous angle was inspected by injecting 7 mL of air. Structures that immediately filled up with air were identified as veins (veins do not have valves in this area) and ligated to ensure pressure could be built up inside the venous reservoir. Air was aspirated from the reservoir by means of a 3-way spigot that was connected between the cannula and the gravity-controlled contrast infusion device. Infusion pressures were maintained for 30 min each, using a 50/50 (*v*/*v*) barium sulphate solution in tap water, instead of dissolving the barium sulphate in pre-vulcanised latex, to examine the influence of controlled, prolonged injection pressure on the patency of the lymphatic valves. Retrograde lymphatic filling was observed in several specimens, but not to a significant extent, usually ending by a rupture of the venous wall or the lymphatic wall, after breaking through the first lymphatic valve. This led to the conclusion that the lymphatic valves were generally better equipped to handle the injection pressure, compared to lymphatic walls or venous walls. The time intervals per injection pressure did not appear to have an added value. If the lymphatic wall or venous wall ruptured, it was usually at the beginning of a new injection pressure cycle, and even if the lymphatics remained intact after the beginning of the pressure cycle, the lymphatic walls or the venous walls still ruptured before the next lymphatic valve did. The lymphatic valves withstood pressures of up to 55 mmHg, while no such pressures were tolerated by the lymphatic walls or the venous walls. Therefore, even though a proof of concept was provided for retrograde lymphatic injections, the experimental set up needed to be optimised further to allow a significant amount of retrograde filling of the subclavian lymphatic trunk [77].

From the previous studies it became clear that retrograde filling of the lymphatics was in fact possible, yet it would require technical adaptations and additional experiments to optimise the experimental procedure. Variables that were identified as being crucial parameters in the previous experiments are listed below. Adaptations to these parameters are expected to further optimise the retrograde injection methodology, to a point where the subclavian lymphatic trunk can be filled up to the axillary lymph nodes.

During the thoracic duct injection experiments it was found that different solvents caused different amounts of lymphangions to retrogradely fill with contrast agent [72], identifying the contrast agent composition as an important variable in the experimental set up. Experimenting with different contrast agents and different solvents could further optimise the experimental procedure. Initially, barium sulphate was chosen as contrast agent, because of previous experience and the good contrast it provides on CT images. To start, the density of lymph was simulated for the contrast solution, assuming that the lymphatics are compatible with this composition. Based on the findings in these experiments, a different train of thought was also explored. Since lymph does not normally move retrogradely, it may not be needed to replicate its characteristics, if a different composition of the contrast agent would be more likely to provide retrograde filling. For example, a fluid with a higher density and viscosity than lymph could exert more pressure on the valves and prove more efficient in breaking through them. When searching the literature after this realisation, a possible alternative contrast agent was found in lipiodol. This contrast agent had previously been used for lymphatic experiments and it was shown to dissolve well in resins that are used for corrosion casting [78]. From experience it was found that hardening contrast agents are preferable to contrast agents that remain liquid. Since corrosion casting can show minute vessels in the same range as the superficial lymphatics [79], this could also be a possibility to look into, keeping in mind that the specimen does not necessarily need to be corroded if the lymphatics are filled with the contrast agent. For this approach, a resin with a sufficiently long hardening time will also need to be identified, since filling the lymphatics will take longer than injecting the arterial and venous system.

The experiments were started in Thiel embalmed specimens, because these specimens are known for their life-like colour and tissue properties and the embalming chemicals prevent putrefaction, allowing an experimental window of up to several years [80]. The Thiel embalming technique is a soft fix embalming techniques that provides a realistic patient model [81,82,83], especially compared to the stiff specimens that are obtained when using formaldehyde-based embalming techniques in an effort to suspend putrefaction and prolong the experimental window. The specimens retain their range of motion, so they can be positioned in treatment positions, thus providing an opportunity to capture the lymphatics in clinically relevant positions, once contrast agent has been injected. The Thiel embalmed specimens provided a good model to standardise the injection technique, but the venous and lymphatic walls proved to be less pressure resistant than the lymphatic valves. When dealing with embalmed specimens, the effects of the embalming chemicals should always be considered. The embalming procedure was shown to alter the micro anatomical tissue structure [84]. The alteration of the micro anatomical structure caused by the embalming procedure could have weakened the lymphatic walls and venous walls and even though Thiel embalmed specimens have been advertised as providing life-like tissue properties [80], biomechanical properties were sometimes found to be altered [85]. On the other hand, the addition of 14 litres of embalming fluid could have also increased the interstitial fluid pressure, thereby causing filling of the lymphatics through the absorbing lymphatic capillaries that normally respond to such rises in interstitial pressure. If the lymphatics have already been filled with embalming fluid and a high interstitial pressure is present, this may complicate the retrograde entry of contrast agent into the lymphatic vessels. As such, it might be worthwhile to experiment with different preservation methods, such as fresh specimens or specimens that are embalmed with a limited volume of embalming fluid, the so-called light embalming technique [86]. Fresh specimens would allow the application of hydrogen peroxide to easily identify the lymphatics [20], while the light embalming technique could produce somewhat firmer specimens, due to an increased amount of formaldehyde in the formulation [80,86]. The much lower volume of embalming fluid used for light embalming (6 L instead of 14) could keep the interstitial pressure low enough for the lymphatics not to be filled, leaving the formaldehyde to interact (firm up) primarily with the lymphatic walls, rather than the lymphatic valves.

Finally, it may not be unreasonable to combine the retrograde injection approach with the anterograde injection approach employed by Suami et al. [20]. If superficial lymphatics could, for instance, be cannulated using this technique and a low-pressure air flow can be introduced, this should open the lymphatic valves. If the retrograde injection is performed at the same time, the denser contrast agent could force the air out of the lymphangion, while the contrast agent can still pass by the lymphatic valve that has not immediately closed yet. All the suggestions above can be tried in combination with each other in order to obtain the ideal conditions that allow for the most efficient retrograde lymphatic injections.

**Figure 4:**
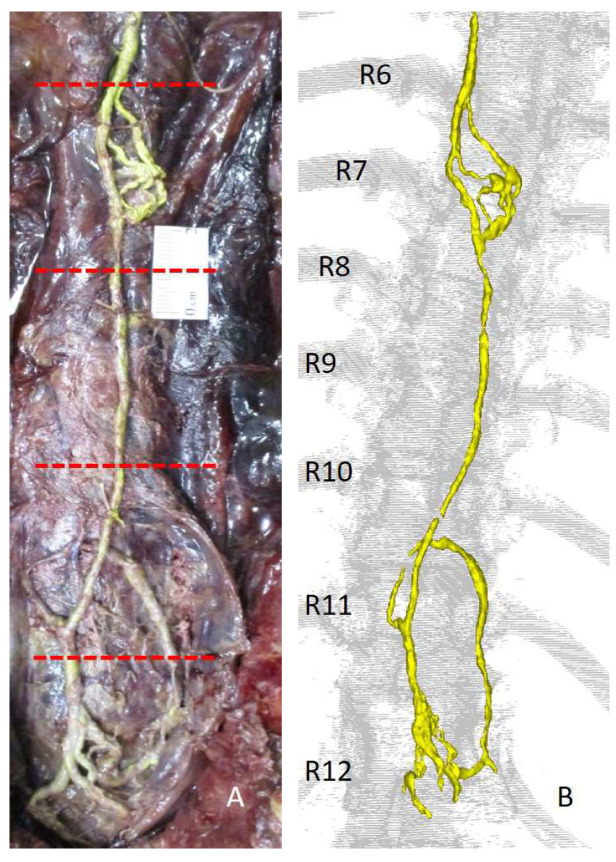
Dissection picture (**A**) and digital reconstruction from the corresponding CT scan (**B**), showing the contrast-filled lymphatics obtained after retrograde lymphatic injection of the thoracic duct. The red dotted lines in figure (**A**) show the possibility to make standardised test units to test retrograde lymphatic injection approaches by ligating the thoracic duct at equally spaced intervals (5 cm). The thoracic duct is shown in yellow. Note how the thoracic duct in the dissection picture and the 3D reconstruction perfectly resemble each other. Also note that this set up provides easy, unobstructed access to the thoracic duct, allowing easy cannulation and visual control of contrast agent advancement during injection. R6–R12 = rib 6–rib 12. Adapted from Stouthandel et al. [72].

The thoracic duct injection methodology is ideally suited to test the above-described experimental approaches in a standardised setting [72]. A good length of thoracic duct is available in this set up that can be ligated at several intervals to form standardised test units within the same specimen. Like this, the test set up is not dependent on different body types (muscle to fat ratio, etc.), or different embalming qualities between bodies and only the impact of the tested parameters will be evaluated. Figure 4 shows the set up for retrograde injection of the thoracic duct after removing the rib cage, the heart and the lungs, with the standardised test units depicted between the red dotted lines. It also shows the possibility to image contrast-filled lymphatics with CT, demonstrating the clear resemblance between the dissection picture and the digital 3D reconstruction.

Assuming that the proposed adaptation to the experimental approach yields the ideal conditions to retrogradely fill the lymphatics, all the way up to the smallest collecting lymphatic vessels, a methodology for capturing these vessels will be required, since they are outside of the spatial resolution of the CT scan that only goes up to 0.2 mm. By combining the regular CT scans with micro CT scans, lymphatic branches up to 0.07 mm in diameter [87] should be visible after reconstructing the contrast-filled lymphatics. To safeguard the spatial relations between the bones (CT) and the smaller lymphatic branches (micro CT), one of our recent studies showed that freezing specimens in treatment position allows the transfer of 3D reconstructed anatomical structures between corresponding datasets, with a submillimetre error margin [88]. By using this technique, the micro CT scans (specimens will need to be sectioned into multiple units to fit in the micro CT scanner) can be imported into the coordinate system of the regular CT scan to provide additional detail to the lymphatic reconstructions, while still presenting the correct spatial relations.

## 3. Molecular Insights in Breast Lymphatics

Tumour metastases at distant sites are the main cause of cancer-related death and about 30% of patients with breast cancer present lymph node metastases, one of the most important prognostic factors in this pathology [89]. The quite recent identification of specific lymphatic markers caused a “renaissance” in the field of lymphatic metastasis research and in the determination of the role of lymphangiogenesis in the metastatic process [52]. Indeed, lymphangiogenesis, analysed by selective lymphatic markers (see below), was found more developed in breast cancer compared to normal breast tissues. Moreover, among the breast tumours, the ones that have metastasised to lymphatics showed higher expression of lymphangiogenesis markers compared to those which were not metastatic [90].

In breast cancer, the chemokine receptors CXCR4 and CCR7 have been involved in the process of the lymph node metastatization and the expression of their ligands CXCL12 and CCL21 by lymphatic endothelial cells attract tumours cells into lymphatic vessels [52,91]. Recently, Xu and colleagues [92] identified and validated a cell subpopulation of breast cancer cells with a high expression level of CXCL14 in the positive lymph nodes of breast cancer patients. The CXCL14 expression was significantly higher in breast cancer patients with lymph node metastasis, suggesting this protein as a new prognostic marker for lymphatic metastasis [92].

Lymphangiogenesis is mainly driven by the VEGFR-3 pathway, with a key role of the endogenous ligands of these receptors such as VEGF-C and VEGF-D [93]. VEGF-C and VEGF-D are expressed in tumour and tumour microenvironment cells, whereas VEGFR-3, a tyrosine kinase receptor, is exposed on the cytoplasmic membranes of lymphatic endothelial cells. Both VEGF-C and VEGF-D promote the formation of intratumour lymphatic vessels binding the VEGFR-3 [94]. The VEGF-C-induced intratumour lymphangiogenesis was associated with the incidence of lymph node metastasis in the preclinical model of metastatization [49]. Interestingly, elevated levels of VEGF-C have been seen in 30–40% of breast cancers and, above all, they are associated with invasion of lymphatic vessels, lymph node metastasis and a shorter period of disease-free progression [50,51].

Lymphangiogenesis could also be a suitable therapeutic target to block lymphatic metastases, but currently no anti-lymphangiogenic compounds have been approved for clinical use. Recently, AD0157, an antiangiogenic drug derived from the marine fungus *Paraconiothyrium* sp., has been proposed as an effective anti-lymphangiogenic compound by García-Caballero and collaborators [95]. AD0157 strongly decreased the tumour-associated lymphangiogenesis and stopped the metastatic dissemination to lymph nodes, by inducing apoptosis in lymphatic endothelial cells and decreasing VEGFR-3 phosphorylation [95].

VEGFR-3 is one of the most used markers for lymphatic endothelial cells in breast cancer tissues, but there are also other proteins reported to be specific for lymphatic vessels such as podoplanin, lymphatic vessel endothelial hyaluronic acid receptor-1 (LYVE-1), and prospero-related homeobox-1 (PROX-1) [96,97]. In particular, podoplanin is the marker usually used to investigate the invasion of lymphatic vessels by breast cancer cells [98]. Recently, Hou and colleagues [99] conducted a large-scale clinical trial to investigate the plasma levels of Hsp90α as a biomarker for breast cancer. Interestingly, they demonstrated that in patients these levels were gradually augmented as the dissemination of cancer cells to regional lymph nodes increased, suggesting a role in lymphatic metastasis for Hsp90α. Moreover, in the same article the authors showed that the administration of recombinant Hsp90α protein in orthotopic breast cancer mouse models enhanced the number of tumour lymphatic vessels and lymphatic metastases, which were blocked by a Hsp90α neutralizing antibody [99]. In vitro, Hsp90α revealed a potent pro-lymphangiogenic characteristic, increasing both the migration and the tube formation of lymphatic endothelium.

In 2019, another promoting factor of lymphangiogenesis and lymph node metastasis in breast cancer was described [100]. Tumour cell-derived lysyl oxidase-like protein 2 (LOXL2) was investigated by immunohistochemistry in tissues from breast cancer patients. LOXL2 was found to be associated with lymphatic vessel density. Moreover, preclinically in mouse models the overexpression of LOXL2 by breast cancer cells significantly enhanced lymphangiogenesis and lymph node metastasis. Of note, LOXL2 was also able to provoke the secretion of the pro-lymphangiogenic factor VEGF-C by fibroblasts [100].

Finally, a number of patient studies have investigated the differences between primary breast tumours and their lymph node metastases in terms of methylation status. Indeed, the methylation pattern of the human deafness, autosomal dominant 5 gene at chromosome 7p15 (DFNA5) increased the risk of lymph node metastasis [101], whereas the hypermethylation of the tumour suppressor gene *CDH1*, which encodes for the transmembrane glycoprotein E-cadherin, increased the axillary lymph node metastasis with worse disease-free survival of these patients [102].

Strategies to limit lymphatic dispersion of cancer have been a focal point of cancer research, in particular after the development of molecular studies on lymphangiogenesis. However, there are no approved drugs that inhibit tumour lymphangiogenesis, despite the well-known consequences of metastasis through the lymphatic system. Consequently, the discovery of new anti-lymphangiogenic drugs is urgently needed in order to respond to a precise therapeutic need that cannot be postponed [103]. Recently, various groups have proposed new compounds that exhibit antilymphangiogenic and antimetastatic properties. Anlotinib, a receptor tyrosine kinase inhibitor, inhibited the onset of metastatic lesions in preclinical and clinical studies through the decrease of lymphangiogenesis and lymphatic metastasis, inactivating VEGFR-3 phosphorylation [104]. Moreover, a fungus-derived molecule named phomaketide A demonstrated to have an inhibitory effect on lymphatic endothelial cells, via VEGFR-3 [105], whereas AD0157 showed a marked anti-lymphangiogenic activity, inducing apoptosis in lymphatic endothelial cells and decreasing VEGFR-3/-2 phosphorylation [95].

## 4. Conclusions

Breast cancer is a major cause of cancer mortality in women. It is one of the most frequently diagnosed neoplastic diseases worldwide and the lymphatic system is the main pathway for spreading of cancer cells. Indeed, lymphatic metastasis is one of the major causes of neoplastic spread in breast cancer patients, because malignant cells colonize lymph nodes and distant organs, leading to worse prognosis [1]. Thus, the knowledge of both anatomical and molecular aspects of the lymphatic network is fundamental to understand the mechanisms of disease progression.

Based on previous experiments, a proof of concept for retrograde lymphatic filling was provided [72,77]. However, many variables play a role in this process and a combination of adaptations to the current experimental approaches is still required to optimise the retrograde filling methodology. The crucial variables that were encountered during the previous experiments are provided and it is expected that by finding the ideal balance between the suggested adaptations, the current methodology can be optimised to provide complete retrograde filling of the subclavian lymphatic trunk.

In terms of future perspectives, by depicting the subclavian lymphatic trunk and its tributaries in detail, in relation to all the surrounding anatomical structures, historical anatomical atlases can be verified and supplemented based on objective spatial relations obtained from the CT coordinate system (rather than from drawings), providing an updated source of clinically relevant lymphatic information. For example, these findings can be used to optimise delineation guidelines for radiation therapy, based on the actual location of the lymphatic system, leading to better tumour control and fewer side effects for the patient. Knowing the exact location of the lymphatic system could also aid surgical resections in the area, by pinpointing common anatomical variations of the lymphatic branches that could be clinically relevant.

Finally, by mapping lymphatic flow patterns in a great number of specimens, tumour spreading could be predicted based on the primary tumour site [106] and physical therapy massages/techniques to treat lymphoedema could be adapted to match lymphatic flow patterns, to promote the natural flow of lymph.

In conclusion, beyond discovering new potent anti-lymphangiogenic drugs for future clinical settings, the deep investigation of breast lymphatics network and lymphatic molecular mechanisms will be essential to support any future development in the treatment of breast cancer.

## Data Availability

Not applicable.
